# Age-dependent patterns of bovine tuberculosis in cattle

**DOI:** 10.1186/1297-9716-44-97

**Published:** 2013-10-16

**Authors:** Ellen Brooks-Pollock, Andrew JK Conlan, Andy P Mitchell, Ruth Blackwell, Trevelyan J McKinley, James LN Wood

**Affiliations:** 1 Disease Dynamics Unit, Department of Veterinary Medicine, University of Cambridge, Madingley Road, Cambridge, CB3 0ES, UK; 2 Animal Health and Veterinary Laboratories Agency, Weybridge, Surrey, UK

## Abstract

Bovine tuberculosis (BTB) is an important livestock disease, seriously impacting cattle industries in both industrialised and pre-industrialised countries. Like TB in other mammals, infection is life long and, if undiagnosed, may progress to disease years after exposure. The risk of disease in humans is highly age-dependent, however in cattle, age-dependent risks have yet to be quantified, largely due to insufficient data and limited diagnostics. Here, we estimate age-specific reactor rates in Great Britain by combining herd-level testing data with spatial movement data from the Cattle Tracing System (CTS). Using a catalytic model, we find strong age dependencies in infection risk and that the probability of detecting infection increases with age. Between 2004 and 2009, infection incidence in cattle fluctuated around 1%. Age-specific incidence increased monotonically until 24–36 months, with cattle aged between 12 and 36 months experiencing the highest rates of infection. Beef and dairy cattle under 24 months experienced similar infection risks, however major differences occurred in older ages. The average reproductive number in cattle was greater than 1 for the years 2004–2009. These methods reveal a consistent pattern of BTB rates with age, across different population structures and testing patterns. The results provide practical insights into BTB epidemiology and control, suggesting that targeting a mass control programme at cattle between 12 and 36 months could be beneficial.

## Introduction

Bovine tuberculosis (BTB) is an infectious disease of cattle caused by the TB species *Mycobacterium bovis*[1]. In Great Britain, the number of cattle slaughtered due to BTB increased from ~200 in 1986 to almost 30 000 in 2010, despite extensive and expensive control measures [2]. Like TB in other mammals, disease is characterised by granulomas that form in the respiratory system from which infectious mycobacteria are excreted [3,4]. However, the focus for control is on the removal of infected cattle at an earlier stage of infection, detected by a positive sensitivity reaction to the single intradermal comparative cervical tuberculin (SICCT) test.

Cattle herds in Great Britain are subject to compulsory regular testing for the purposes of both surveillance and control [5]. Herds in the highest incidence areas have all cattle over 6 weeks tested annually, whereas herds in low incidence areas must test all cows and male breeders every four years [6]. Reactor cattle (cattle that react to the SICCT test) are slaughtered and the breakdown herd is subject to movement restrictions until a number of follow-up tests are passed. Although the surveillance system generates a wealth of data, it has not been possible (until very recently) to estimate age-specific BTB risks because negative results were systematically recorded at the herd level only.

Quantifying the age-specific risk is a key tool for estimating critical vaccination thresholds and the basic reproductive ratio of a disease, *R*_0_[7,8]. Once an epidemic has reached a steady state, *R*_0_ is given by the ratio of the distribution of life times to the distribution of ages at first infection [9]. The theory behind disease control is to reduce *R*_0_, which results in an older average age of infection. An age-varying risk of infection can be exploited for control purposes to reduce the effort required for eradication [7]. For instance, if infection rates in calves were higher than in older cattle, then increasing the average age of infection would have a bigger impact than expected under the assumption of a risk that is independent of age.

Age-stratified serological surveys have been used to estimate the age-specific risk of infection for many childhood diseases [10]. Age-stratified case reports can also be used, provided that two conditions hold: that all individuals experience infection during their lifetimes and that there are no age-related differences in detection [7]. For BTB in Great Britain, only a small percentage of animals are infected during their lifetimes and the age-related tested patterns are unknown, so that without additional data, reactor reports alone cannot be used to quantify the risk of infection with age. In this paper, we combine herd-level test data and spatial movement data from the Cattle Tracing System (CTS) to estimate background testing patterns in order to provide estimates of age- and time-dependent BTB risks in cattle. We use the age-specific reactor rates to parameterise a general age-structured catalytic model of BTB infection in order to explore the patterns with age and impact of testing.

## Materials and methods

### Data

Great Britain has a rich BTB dataset dating back to the 1950s when the first test-and-slaughter scheme was introduced to control disease [11]. Herd-level test results for test-negative herds and animal-level test results for reactor cattle, inconclusive reactor cattle and tests resulting from contact tracing are contained in the database VetNet, collated and managed by the Animal Health and Veterinary Laboratories Agency (AHVLA), which is part of the UK department for Food, the Environment and Rural Affairs (Defra).

Cattle demographic and movement data are contained within Cattle Tracing System (CTS). Introduced in 1996, the CTS contains the births, movements and deaths of all registered cattle in Great Britain, cattle data (sex, breed) and location data [12]. The CTS is run by the British Cattle Movement Service (BCMS), also part of Defra. We used an extract from VetNet covering BTB tests between 2004 and 2009 and a CTS extract for the same period. The data were provided by Defra via the AHVLA and RADAR (Rapid Analysis and Detection of Animal-related Risks). We collated the data using PostgreSQL [13] and carried out the modelling in R [14].

### Herd test types

All herds in GB are subject to regular SICCT testing at a frequency determined by the local incidence of infection [6,15]. Herds in annual testing areas have a whole herd test (WHT) once a year of all cattle over 6 weeks that are present on the date of the test. Herds in four-yearly testing areas are subject to a routine herd test (RHT) once every 4 years of female animals that have calved, male breeders and male calves intended for breeding that have been purchased since the last test. Herds with confirmed infection at a surveillance test are subject to two short interval (SI) tests at 60 days and 120 days, then additional follow-up tests after 6 months (6M) and twelve months (12M). For a more detailed description of the testing programme, see [6,15].

### Reactor data

The number of reactors was collated directly from the “animal” table in VetNet. Reactor cattle were identified by the test result “R”. Inconclusive reactors or other results were not included. Reactors were classified by age in whole years (test date minus birth date), sex (M or F), calendar year of test, purpose (beef, dairy or mixed) based on breed and test type (routine herd tests (RHT), whole herd tests (WHT), short-interval tests following a breakdown (SI) and follow-up tests at 6 months (6M) and 12 months (12 M) after passing breakdown release).

### Inferring negative tests

In general, there is no historic information in VetNet about cattle that tested negative to the SICCT test. In order to estimate underlying testing patterns, we reconstructed the herd on the test date by combining herd-level testing data from VetNet with animal-level data from the CTS. From the list of cattle that were present during a test, we used Defra’s eligibility guidelines to determined which of the cattle had been eligible for the test. We assumed that all eligible present animals were tested. In detail, we extracted the date on which herds were tested and the type of test that was carried out from the VetNet testing table. If testing took place over several days and was recorded multiple times in VetNet, we took the date of the first day of the test. Then the CPH (county-parish-holding) number was matched to the CTS locations table to obtain a CTS id number. Using the CTS id number, we extracted a list of cattle id numbers that had moved onto the premises before the test date and left after the test date (or had not left at all) from the CTS movement table. The cattle id numbers were used to determine the sex and age at the time of the test. We determined eligibility according to the type of test that was carried out:

•Whole herd tests (WHT) and follow-up tests (SI, 6M, 12M): all animals aged over 6 weeks.

•Routine herd tests (RHT): female animals that have calved, male breeders, male calves intended for breeding that have been purchased since the last test.

Female animals that had calved and male breeders were identified using the relationships data in the standard CTS extract and, as the relationships data were not complete, by the age of the animal. It was difficult to identify male calves intended for breeding, so instead we included male animals that had yet to be tested in that herd. Between 2004 and 2009, 700 979 out of 701 577 VetNet records matched a CTS record. In terms of premises, 87 177 distinct CPH numbers were recorded as having a test between 2004 and 2009. We were able to match 92,251 (99.6%) in the CTS location table. Of the cattle records, 1.4% were removed due to missing birth dates.

Once a list of cattle had been obtained, we classified the tested cattle (as for reactors) by age, sex, purpose and tests by the test type and calendar year.

### Reactor rates

We used the number of reactors found during herd tests (WHT, WHT2, RHT, SI, 6M, 12M) and the estimated number of cattle tests carried during those tests to calculate the positivity or reactor rate by age and purpose based on breed type, *r*(*a*), equation (1).

(1) $$r\left(a\right)=\frac{\text{Number}\;\text{of}\;\text{reactors}\;\text{aged}\;a}{\text{Number}\;\text{of}\;\text{cattle}\;\text{tested}\;\text{aged}\;a}$$

Because the denominator is the number of cattle tested not the total cattle population, the reactor rates are effectively weighted towards areas with higher levels of testing. Given the imperfect sensitivity and specificity of the SICCT test, the reactor rates only approximate true prevalence. However, as reactor rates are relative low (≤ 2.5%), the discrepancy is negligible and does not impact on the overall results [16].

### Other demographic data

Collated mortality data were extracted from the CTS livestock table by calendar year, age, sex and breed purpose (beef or dairy).

### Modelling BTB

To capture the dynamics of cattle becoming infected and detected with BTB with age, we used a simple two-state catalytic model [8]. A catalytic model is similar to a transmission model but without including an explicit mechanism for transmission [17], instead relying on a set of force-of-infection parameters. Cattle were assumed to be not infected and non-reactors (state *S*) or infected (state *I*). Infected cattle were tested and removed from the system as reactors. The basic age (*a*) dependent equations governing each infection state are

(2) $$\frac{\mathrm{dS}\left(a\right)}{\mathrm{dt}}=-\lambda \left(a\right)S\left(a\right)-\mu \left(a\right)S\left(a\right)$$

(3) $$\frac{\mathrm{dI}\left(a\right)}{\mathrm{da}}=\lambda \left(a\right)S\left(a\right)-\mu \left(a\right)I\left(a\right)-\mathrm{\gamma I}\left(a\right)$$

where *λ*(*a*)is the age-dependent force-of-infection, *μ*(*a*) is the age-dependent death rate and *γ* is the removal rate of infected animals due to testing. The number of reactors aged *a* is given by *γI*(*a*). This formulation assumes that the force-of-infection and other time-varying parameters do not vary significantly over the course of a year and do not depend on time. Integrating equations (2) and (3) with respect to age gives equations for the number of cattle in each infection state for each age, equations (4) and (5):

(4) $$S\left(a\right)\;=\;\text{exp}\;\left(-\int_{0}^{a}\left(\lambda \left(x\right)+\mu \left(x\right)\right)\mathrm{dx}\right)$$

(5) $$\begin{array}{l} I\left(a\right)\;=\;\text{exp}\;\left(-\int_{0}^{a}\left(\gamma +\mu \left(x\right)\right)\mathrm{dx}\right)\\ \;\times \left(\int_{0}^{a}\lambda \left(a\right)\text{exp}\left(-\int_{0}^{x}\left(\lambda \left(b\right)-\gamma \right)\mathrm{db}\right)\mathrm{dx}\right)\cdot \end{array}$$

We make a standard simplification that the population is divided into discrete age cohorts [10]. We define the *i*^th^ cohort as containing cattle aged between *i* and (*i +* 1) years. The rates governing transitions between states become piecewise functions: for cattle in cohort *i*, *λ*_*i*_ is the age-specific force-of-infection or infection risk (to be estimated) and *μ*_*i*_ is the age-specific mortality rate (calculated from the data). Therefore, the number of cattle in each discrete cohort is given by equations (6) and (7):

(6) $$S_{i}=\int_{i}^{i+1}S\left(a\right)\mathrm{da}=\Psi_{i}\left(\lambda +\mu \right)$$

(7) $$I_{i}=\int_{i}^{i+1}I\left(a\right)\mathrm{da}=H\left(i\right)\Psi_{i}\left(\gamma +\mu \right)-\frac{\lambda_{i}}{\lambda_{i}-\gamma }\Psi_{i}\left(\lambda +\mu \right)$$

where Ψ_*i*_ is the difference of exponential functions

$$\Psi_{i}\left(\mu \right)\;=\;\frac{\exp\;\left(-\sum_{x=0}^{i-1}\mu_{x}\right)-\exp\sum_{x=0}^{i}\mu_{x}}{\mu_{i}}$$

and

$$H\left(i\right)=\frac{\lambda_{0}}{\lambda_{0}-\gamma }+\sum_{k=1}^{i}\frac{\lambda_{k}}{\lambda_{k}-\gamma }-\frac{\lambda_{k-1}}{\lambda_{k-1}-\gamma }\;\exp\;\left(-\sum_{j=0}^{k-1}\lambda_{j}=\gamma \right)$$

Full derivations of equations (6) and (7) are in the Additional file 1 and further details of similar derivations can be found in [8] (Appendix D, pages 677–678), with the difference that we explicitly include mortality as the total population size decreases due to loss of reactors. Initial conditions are *S*_0_ = 1, *I*_0_ = 0, and for the other age groups the initial conditions are computed from the equations.

We investigated two alternative formulations of *λ*_*i*_: one where *λ*_*i*_ is independent of age but dependent on calendar year such that *λ*_*iy*_ *= β*_*y*_, and one in where the effects of age and year are separable and distinguishable, such that *λ*_*iy*_ *= α*_*i*_*β*_*y*_.

From these age-specific equations, we calculate the average age at infection as $A_{I}=\sum_{i}i\lambda_{i}S_{i}/\sum_{i}\lambda_{i}S_{i}$ and the average age at detection as $A_{D}=\sum_{i}\mathrm{i\gamma}I_{i}/\sum_{i}\gamma I_{i}$. A lower bound for the average number of secondary cases per infected animal (*R*_ƒ_) is given by the reciprocal at the proportion of susceptible cattle, calculated as $R_{f}=\sum_{i}N_{i}/\sum_{i}S_{i}$.

### Parameter estimation

The age-specific mortality rates were calculated from the CTS. We estimated the removal rate and the force-of-infection for each age group and year in a Bayesian framework using Markov chain Monte Carlo (MCMC), implemented with the MCMCpack library in R [18]. For each year *y*, the probability that *K*_*iy*_ cattle react to the test if *T*_*iy*_ cattle in cohort *i* are tested follows a binomial distribution, and so the likelihood is equation (8):

(8) $$L=\prod_{i,y}\left(\begin{array}{c} T_{\mathrm{iy}}\\ K_{\mathrm{iy}} \end{array}\right)p_{\mathrm{iy}}^{K_{\mathrm{iy}}}{\left(1-p_{\mathrm{iy}}\right)}^{\left(T_{\mathrm{iy}}-K_{\mathrm{iy}}\right)}$$

where *p*_*iy*_ = *γI*_*iy*_/(*S*_*iy*_ + *I*_*iy*_) is calculated using the model (equations 6 and 7) as a function of *λ*_*iy*_. To improve convergence for *λ*, we re-parameterised the model to estimate log (*λ*) and sampled from a flat prior *log*([1*e*^− 10^, 1*e*^− 1^]). We used the *optim* function to obtain maximum likelihood estimates for the parameters that were used as starting points for the MCMC chain. Following visual inspection of the MCMC trace plots, we generated 10 000 samples of the posterior distributions from a chain of 40 000 samples (i.e. sampling every 4^th^ sample) and with a burn-in of 10 000 samples. We checked convergence and mixing with a visual inspection of the MCMC trace plots and model fit and by comparing multiple MCMC chains.

We compared the two formulations for *λ*_*iy*_ (year dependent and age and year dependent) using the Bayesian Information Criterion (BIC), which is defined as (equation (9)):

(9) $$-2\log\left(L\right)+n\log\left(k\right)$$

Where *n* is the number of parameters and where *k* is the number of observations and log (*L*) is the mean log likelihood of the MCMC chain. We identified the model with the largest explanatory power by the lowest BIC score.

### Impact of removal rate

Infected cattle are removed in the model via testing and death only (there is no recovery or death due to infection). In fitting the model, we found that the removal rate was highly correlated with the force-of-infection experienced by the first cohort and therefore could not be estimated independently. Using an unconstrained prior allowed solutions in which the removal rate was close to zero, which is not believed to be the case [19,20]. Therefore, we used an informative prior for the removal rate with distribution ~ *N* (0.6, 0.02)which led to posterior estimates ranging from 50% to 70%. Furthermore, we tested the sensitivity of the results to the removal rate. As is discussed in the results, the removal rate affects the magnitude of the *λ*_*i*_s for young cattle but not the patterns with age. For model simplicity, we did not include variable sensitivity and specificity associated with different test interpretations [19,21], age-specific test sensitivity or any desensitisation associated with repeated testing.

## Results

### Demography and patterns of cattle testing

**Figure 1:**
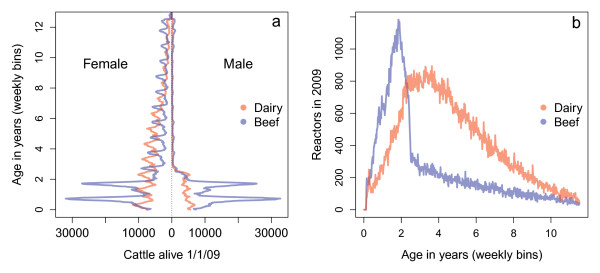
**Demographics of the cattle population in Great Britain. a)** Population pyramids for dairy and beef cattle in Great Britain. The distributions were calculated using the Cattle Tracing System on 01-01-2009. **b)** Unadjusted age-specific reactor numbers for 2009 for dairy and beef cattle in Great Britain, calculated from the VetNet database.

The age structure of the cattle population remained relatively stable between 2004 and 2009. The significant differences between the dairy and beef populations can be seen in the age pyramids in Figure 1a. In 2009, the 9 million cattle population was made up of 34% female beef, 31% female dairy, 19% male beef and 7.4% male dairy animals. Contrasting calving patterns between dairy and beef result in alternating peaks in age groups. Of the 35% of cattle that live for three or more years, 48% are female dairy and 40% are female beef animals. The turnover of the beef population is just over 30% per annum, whereas in the dairy stock turnover is just under 10%. The distribution of reactors by age (Figure 1b) is noticeably different for dairy and beef cattle. In 2004, the mean age of dairy reactor cattle was 4.72 years and this decreased to 4.60 years by 2009. For beef cattle the average reactor age dropped from 3.41 years to 3.37 years.

Between 2004 and 2009, the number of cattle tests increased by 28%. Combining VetNet and the CTS, we find that the age distribution of tests remained largely consistent over that period, with on average 66% of tests conducted on cattle 3 years or younger (Additional file 2). In 2005, the number of tests of beef cattle exceeded the number of tests on dairy cattle and remained higher for the following years. Beef cattle are on average tested at younger ages compared to dairy cattle (37 months versus 48 months). We found that January born animals of all breeds were tested at older ages than animals born in other months (50 months versus 41 months).

### Age-specific reactor rates

**Figure 2:**
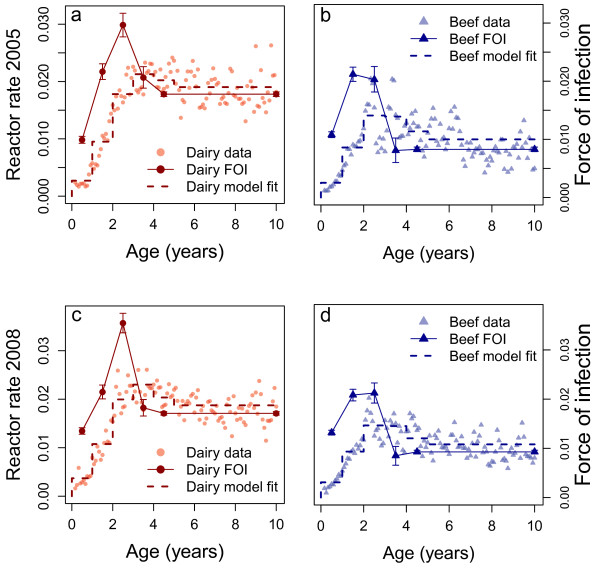
**Age specific reactor rates and estimated force of infection.** Panels **a)** and **b)** show data and fits for the age and year model for 2005; panels **c)** and **d)** for 2008 for dairy (panels **a** and **c**) and beef (panels **b** and **d**) cattle. In each panel, age-specific reactor rates are given by the points; the joined points with error bars show the mean estimated age-specific force of infection with 95% credible intervals and the step function shows the model fit using the mean force of infection for each cohort.

Normalising reactor numbers by age-specific testing patterns revealed a clear association between age and reactor rates (Figure 2a-d and Additional files 3, 4 and 5). The general pattern is typical of age-specific rates for other infectious diseases, including TB in humans. This provides good grounding for the use of an SI-type catalytic model. Reactor rates increased with age for the first 2–3 years of life, after which rates in older animals remained constant or decreased. Dairy cattle over 2 years experienced 40% higher rates than beef cattle of similar age but notably, the reactor rates in young beef and dairy calves under 1 year are approximately equal. Separated by test type, short interval (SI) tests have the highest reactor rates, followed by follow-up tests, and whole herd tests (WHT). The lowest reactor rates are seen during routine herd tests (RHT) (Additional file 3).

Although the number of tests increased year on year between 2004 and 2009, we find elevated reactor rates in 2005, 2008 and 2009. The lowest reactor rates were in 2006 when we estimate that 0.73% of tests were failed tests. Although there were over 7000 more reactors in 2009 compared to 2005, we estimate that the reactor rates were equal up to two significant figures (1.12% in 2005 compared to 1.10% in 2009).

### Age-specific catalytic model

Fitting the *λ*_*jy*_s independently, we estimate a force-of-infection ranging between 0.003 years^-1^ (beef cattle aged 3–4 years in 2006) and 0.036 years^-1^ (dairy cattle aged 2–3 years during 2008). For both dairy and beef cattle and across all years, the highest rates of infection were in cattle aged 2–4 years.

Infection rates for cohort 0 (0 to 1 years) were similar for dairy and beef cattle across all years, suggesting that these youngest cattle experience similar risks despite being located in different parts of the country and in different herd structures. Infection rates for cohort 0 increased from 0.0083 years^-1^ in 2004 to a maximum of 0.013 years^-1^ in 2008. The infection risk in these youngest cattle may reflect true infection annual risk, as they will not have experienced infection in previous years.

In cohort 4, at 3 years, the number of cattle removed as reactors exceeds the number of new infections generated. In ages up to 3 years, the imperfect removal rate results in infected cattle left behind in the population. After 3 years, the number of new infections is less than the number of reactors and residual infection is cleared. The differential impact of removal rate with age is reflected in the 2d posterior density plots (Additional file 6). In cohort 0, there is a strong co-dependence between infection rate and removal rate, where higher removal rates are balanced by lower infections rates, and vice versa, higher infection rates can be balanced by a lower removal rate to produce equally good model fits. However, after 3 years, the trade-off between infection and removal rate is no longer observed and the infection rates are well defined.

**Figure 3:**
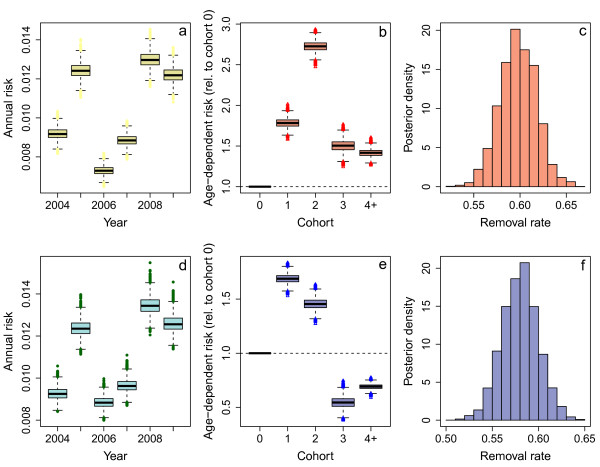
**Results and parameter estimates from the age and year-dependent model.** Panels **a)** and **d)** show the year-dependent infection rates for dairy (red) and beef cattle (blue). The box represents the central 50% of the posterior distribution with the median marked by the central line. The outer whiskers represent the 95% CI and the outliers are shown as points. Panels **b)** and **e)** show the age-dependent infection risks for dairy and beef cattle. Panels **c)** and **f)** give the posterior density for the removal rate for dairy and beef.

The consistent shape of the age profiles across the years suggested an underlying age-dependent process. Using an age-independent infection rate gave a BIC score of 5019 for dairy cattle and 4694 for beef cattle. Including an age-dependent risk significantly improved model fit for both dairy and beef populations (BIC 1139 for dairy cattle and 1407 for beef cattle), indicating a strong age-dependent signature in the data. Model fits for the age-dependent model are shown in Figure 3a-f and supplementary Figures 3 and 4. Figure 3a and d show the annual risk for dairy and beef cattle. Both show very similar patterns of increased risk in 2005, 2008 and 2009.

**Figure 4:**
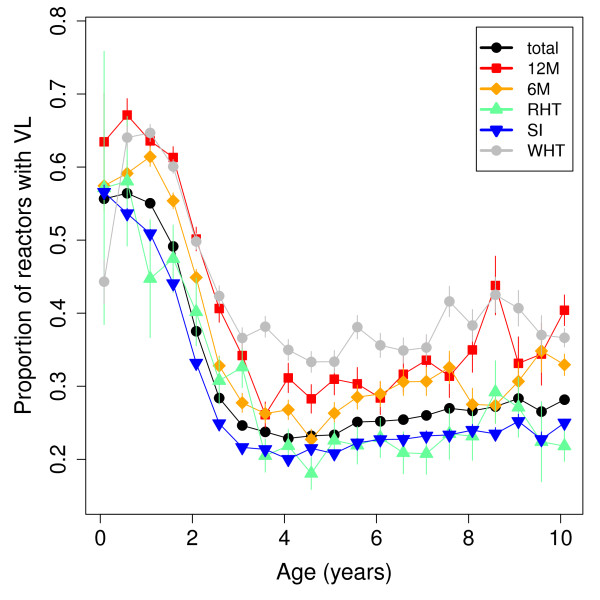
**The relationship between reactor age and probability of finding visible lesions (VL) at slaughter.** The different coloured lines show the results from different test types. The data were extracted from VetNet.

The age-dependent risks were different for dairy and beef cattle. In dairy cattle (Figure 3b), all ages had an increased risk of reacting relative to cohort 0, with the greatest relative risk experienced by cattle aged 1–2 years. In beef cattle (Figure 3e), cattle aged 1 to 2 years experienced a greater risk, but then cattle over 3 years experienced a lower risk than cattle under 1 year.

These estimates for force-of-infection can be interpreted in terms of cattle ages. We estimate that the mean age of infection in dairy cattle was 4.4 (3.9, 4.6) years. The mean age at detection over the same period was 8 months later at 5.1 years. The mean percentage of susceptible dairy cattle was 97.9% (calculated using equation 6), which suggests that the reproductive number in the population was not less than 1.03. In beef cattle, the mean age at detection was 3.8 years, but we estimate that the mean age at infection was 3.3 years. In beef cattle, the mean proportion of susceptible animals was 98.6%, again suggesting a reproductive number of not less than 1.015.

## Discussion

To our knowledge, this work is the first attempt to characterize the age-specific dynamics of TB in cattle. We demonstrated that the age-specific reactor rates cannot be explained by a cumulative constant hazard over an animal’s lifetime. Knowledge of age-specific risks is essential for accurately interpreting trends in pooled epidemiological data and for the design and assessment of control strategies such as vaccination.

The method we describe here provides a way of estimating age-specific reactor rates using herd-level testing data combined with cattle movement records and can be applied at a local or national scale. Combining the detailed spatial demography contained in the Cattle Tracing System with the national epidemiological data in VetNet has provided insight into BTB epidemiology and control in Great Britain, although there is an important distinction between test results and true infection rates. As the majority of tests are conducted in high incidence areas in Britain, the results presented here reflect disease dynamics in high incidence regions. In low incidence settings, beef cattle intended for slaughter are not routinely tested, and therefore are not fully captured here. Mitchell et al. demonstrated that many cattle, particularly in low incidence areas, are never tested during their lifetimes because of turnover and movement between herds [22]. Indeed, this is reflected by the low numbers of young animals tested during routine herd tests. We believe that the increase in incidence with age reflects an intrinsic property of bTB transmission. However, to estimate the magnitude of the force-of-infection experienced by a different population, it would be necessary to measure both reactor numbers and underlying testing patterns with age.

This analysis is based on routinely collected surveillance data does not address the underlying immunology of BTB infection, nor attempt to capture the complex BTB transmission process. Our motivation was to obtain a parsimonious interpretation of the available data without assumptions about the relationship between test status and infectiousness or the interaction between infected cattle, infected wildlife and the local environment. Previous approaches to modelling BTB have included multiple stages of infection [23-25], including a latent or occult period immediately following infection when infected animals may be unresponsive to the skin test. Including an occult period in our model formulation would increase the estimated infection risks as a higher proportion of infected cattle would die before being detected. The natural next step for this work is to include an explicit transmission process to capture the contact process between susceptible and infectious cattle in a “who acquires infection from whom?” framework. We did not include an explicit transmission process in this model because of the complex and fiercely debated role of badgers in cattle transmission dynamics.

Nevertheless, the substantial variation of BTB rates with age has implications for both the understanding of BTB infection and the design and implementation of control programmes. With TB in humans, the tuberculin skin test has a lower sensitivity for disease in young children than in other age groups [26] and it has been suggested that vaccinating neonates may be suboptimal due to differential response to infection. Comparison can also be drawn with *Mycobacterium avium* subspecies paratuberculosis (MAP) infection in cattle. Response to MAP has been shown to vary with age with young calves at increased risk of developing high bacterial loads [27,28]. Extrapolating from TB and MAP to BTB, it could be that young calves have a lower chance of detection but have the potential to be high shedders. Further analysis of VetNet indicates that 50-60% of reactors under 2 years are found with visible lesions, compared to around 30% in older reactors (Figure 4). Further work is needed to quantify the role of calves and older cattle in the transmission process.

Many studies have found differences in BTB risk between dairy and beef cattle [29-31], and indeed the unadjusted age profiles of beef and dairy reactor numbers are hugely different (Figure 1b). However, we found that when reactor numbers are normalised by test patterns the age dependencies are remarkably comparable. In fact, we found that beef calves under 1 year experience similar risks of reacting to dairy cattle of the same age. This highlights interesting questions about the drivers of the differences between older beef and dairy cattle and the interaction between breed, herd structure and age. Could the differential risks in age and breed be exploited for improved control? A detailed understanding of age-dependent patterns in different countries with different infection burdens will help to elucidate the true characteristics of BTB infection in cattle.

## Abbreviations

TB: Tuberculosis; BTB: Bovine tuberculosis; SICCT test: Single intradermal comparative cervical tuberculin; CTS: Cattle Tracing System.

## Competing interests

The authors declare that they have no competing interests.

## Authors’ contributions

EBP conceived the study and was involved in all aspects of the work; AJKC contributed to developing the mathematical model and parameterisation; APM and RB were involved with data processing and analysis; TJM contributed to statistical methodology; JLNW advised on direction and interpretation of results; all authors contributed to the interpretation of results and read and approved the final manuscript.

## Supplementary Material

Additional file 1 **Detailed derivations of the equations used in the manuscript.** This file contains derivations of the exact solutions for *S*_*i*_ and *I*_*i*_. Click here for file

Additional file 2 **Distribution of tests by age and breed.** Figure showing the number of SICCT tests by age and breed purpose for 2009. The herd-level tests included are: whole herd tests in annual and biannual testing areas, routine herd tests, short interval tests, 6 month and 12 month follow-up tests. Click here for file

Additional file 3 **Reactor rates for the SICCT test by age and test type.** Figure showing reactor rates to the SICCT test by age and test type. The test types are WHT: Whole herd test, RHT: Routine herd test, SI: Short interval test and 12 M: 12 month follow-up tests. Click here for file

Additional files 4 **Model fits for dairy cattle for the years 2004 to 2009.** Figures show data and model fit for the age/year-dependent model for dairy cattle between 2004 and 2009. In each panel, the points are the data and the step function is the model fit from the age and year dependent model. Click here for file

Additional file 5 **Model fits for beef cattle for the years 2004 to 2009.** Figures show data and model fit for the age/year-dependent model for beef cattle between 2004 and 2009. In each panel, the points are the data and the step function is the model fit from the age and year dependent model. Click here for file

Additional file 6 **2D posterior densities illustrating the relationship between infection and removal rates.** Figure illustrating the relationship between the removal rate and the age-specific infection rate. The areas of greatest probability are illustrated in red and lowest probability in blue. The force-of-infection (FOI) for each cohort is shown on the vertical axis and the removal rate on the horizontal axis. Click here for file
