# Supplementary material for: Age-dependent patterns of bovine tuberculosis in cattle
Source: Vet Res. 2013 Oct 16;44(1):97. doi: 10.1186/1297-9716-44-97 (PMC3853322; doi:10.1186/1297-9716-44-97)
Supplement: Additional file 1 — Detailed derivations of the equations used in the manuscript. This file contains derivations of the exact solutions for Si and Ii. [file 1297-9716-44-97-S1.doc]

**Additional file 1 Derivation of main equations.**

*Exact solution for*
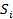


Following on from the main paper, the equation for $dS/da$ is

In the discrete case with $n$ age cohorts, we have that $\lambda(a)=\lambda_{i}$, $\mu(a)=\mu_{i}$ and $\gamma(a)=\gamma_{i}$ for $a\in[i,i+1)$. The number of susceptible animals in cohort $i$ is

Integrating equation 1 gives

For $\max(i)$s.t. $a\leq(i+1)$ and $i\in Z$,

So,

Therefore, the number of susceptibles in age group $i$ is

Writing

then

*Exact solution for*
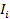


In the model, infected cattle are removed at rate $\mu(a)+\gamma(a)$ from the system. The equation for $dI/da$is

Using the appropriate integrating factor,

then substituting for $S(a)$, the $\mu$terms on the RHS cancel, giving

If $i$ is the largest integer such that $a\leq(i+1)$

Firstly consider the integral over al single cohort

Then,

The remaining integral is

The LHS of equation 2 equals

So, the full expression for $I(a)$ is

This has two terms involving $a$,

where

So,

Finally,

where $H_{i}=H'(i)+K(i)$ and as

Re-writing $H(i)$ gives

As a check, it is easy to see that when there is no removal and $\gamma(a)=0,\text{ }H(i)=1$ and
