# Supplementary figures and images for: Age-dependent patterns of bovine tuberculosis in cattle
Source: Vet Res. 2013 Oct 16;44(1):97. doi: 10.1186/1297-9716-44-97 (PMC3853322; doi:10.1186/1297-9716-44-97)

Number of cattle tests 2009

● Dairy  
● Beef

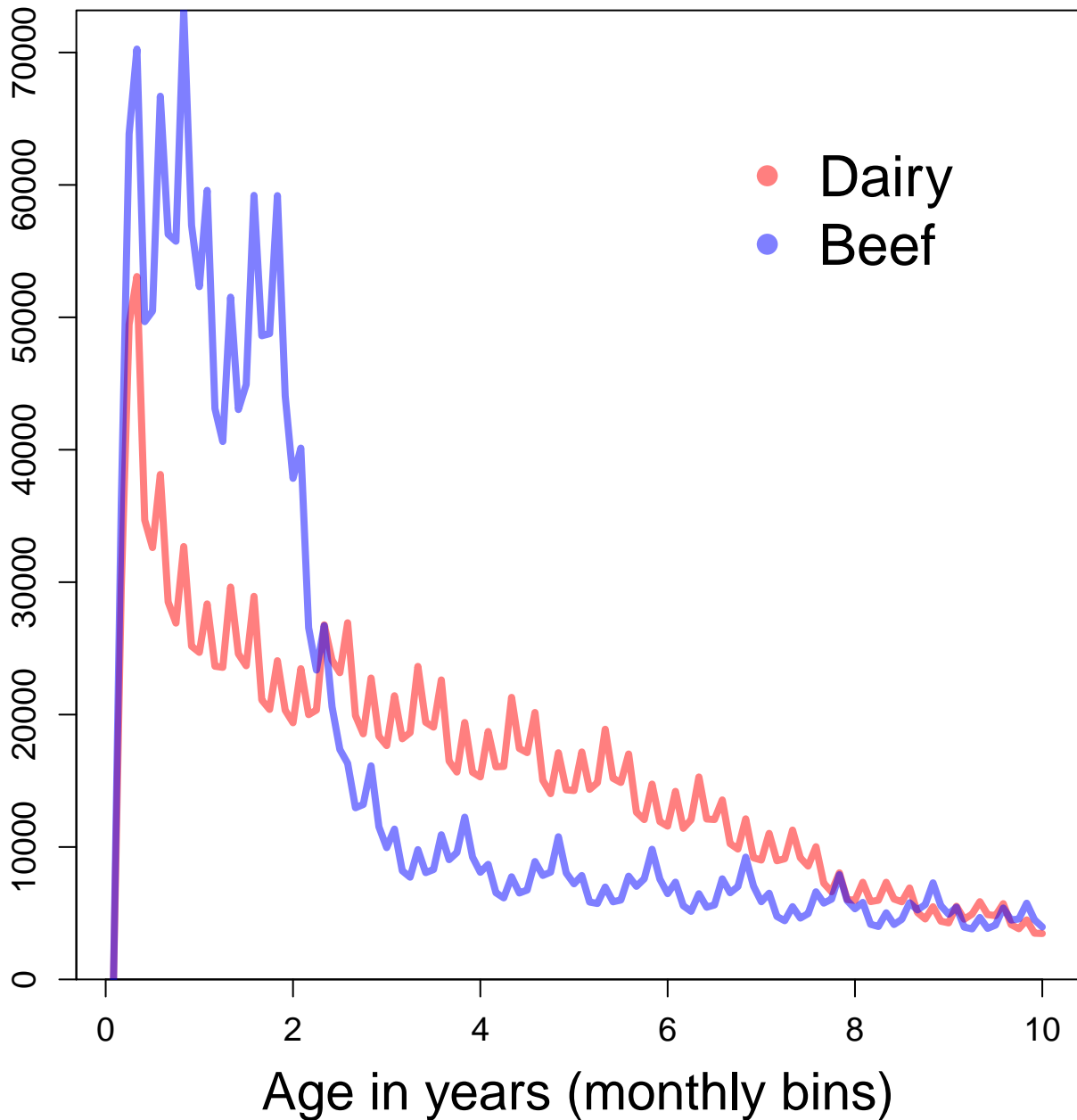

Supplement: Additional file 2 — Distribution of tests by age and breed. Figure showing the number of SICCT tests by age and breed purpose for 2009. The herd-level tests included are: whole herd tests in annual and biannual testing areas, routine herd tests, short interval tests, 6 month and 12 month follow-up tests. [file 1297-9716-44-97-S2.pdf]

% cattle testing positive

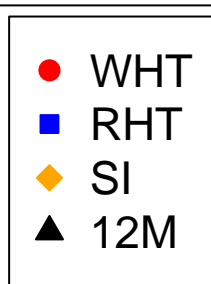

0 20 40 60 80 100 120

Age (months)

2.5  
2.0  
1.5  
1.0  
0.5  
0.0

Supplement: Additional file 3 — Reactor rates for the SICCT test by age and test type. Figure showing reactor rates to the SICCT test by age and test type. The test types are WHT: Whole herd test, RHT: Routine herd test, SI: Short interval test and 12 M: 12 month follow-up tests. [file 1297-9716-44-97-S3.pdf]

**2004**

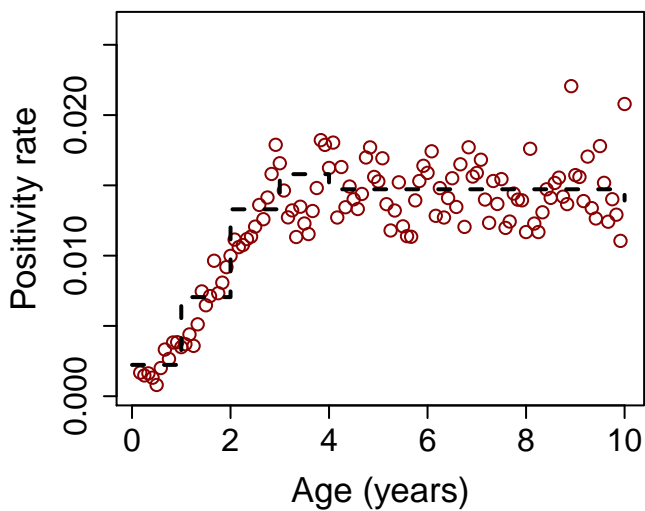

**2005**

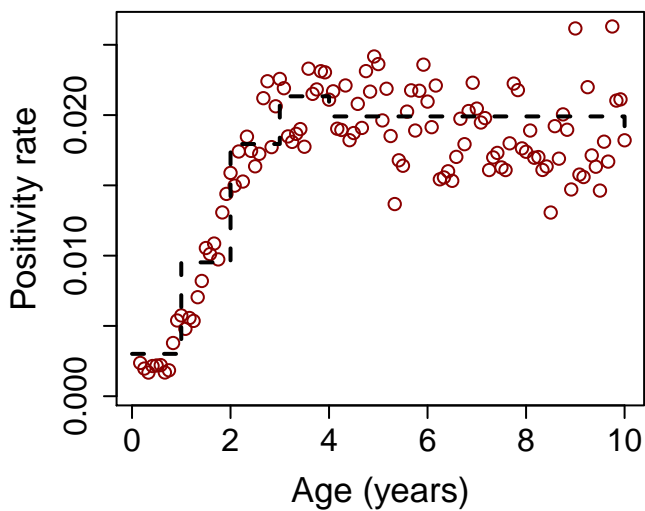

**2006**

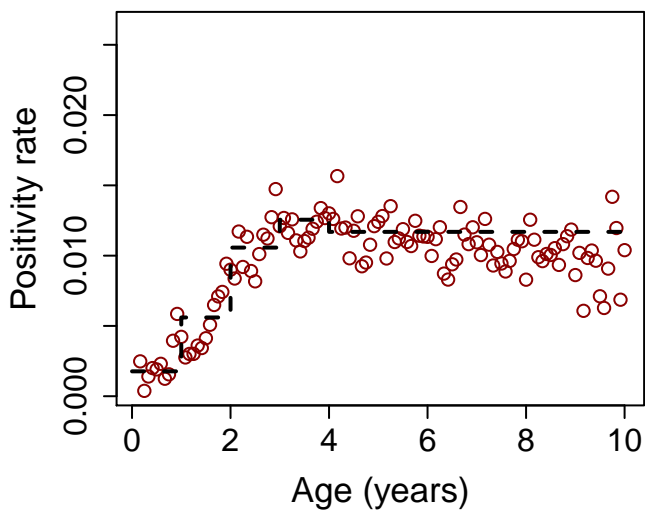

**2007**

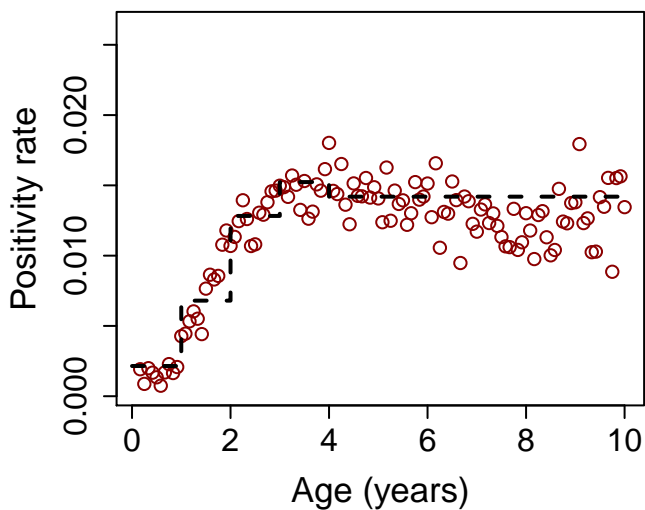

**2008**

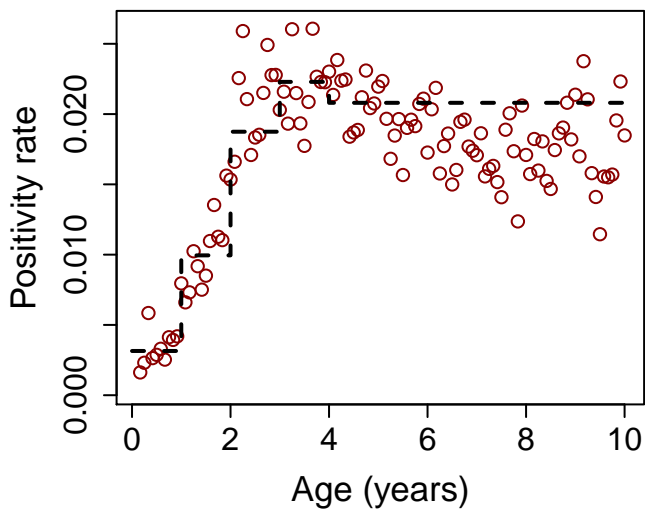

**2009**

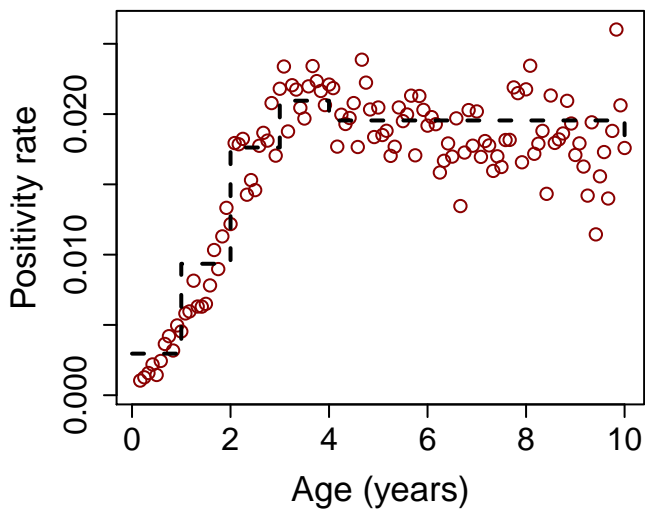

Supplement: Additional files 4 — Model fits for dairy cattle for the years 2004 to 2009. Figures show data and model fit for the age/year-dependent model for dairy cattle between 2004 and 2009. In each panel, the points are the data and the step function is the model fit from the age and year dependent model. [file 1297-9716-44-97-S4.pdf]

**2004**

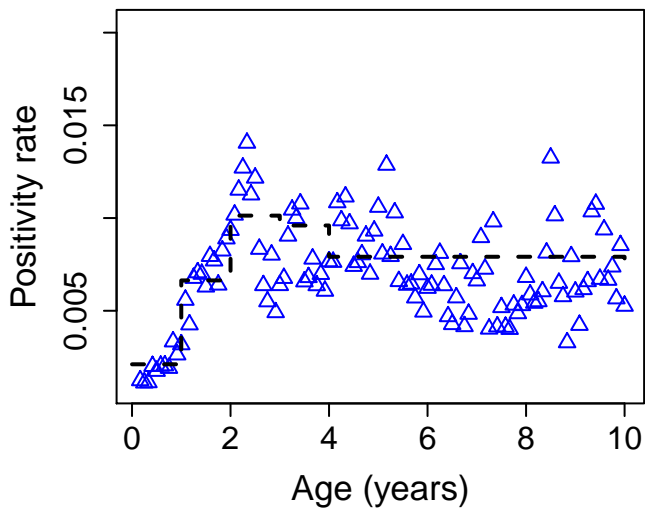

**2005**

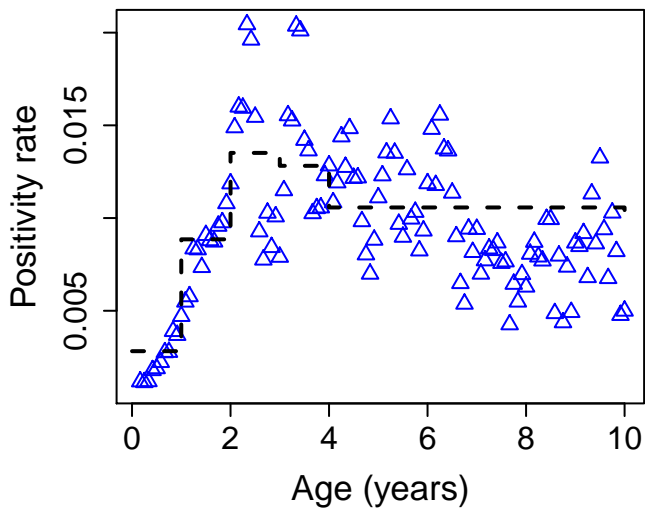

**2006**

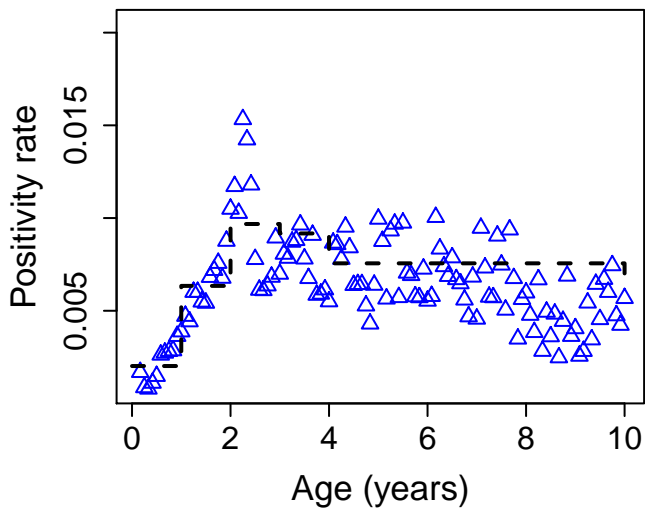

**2007**

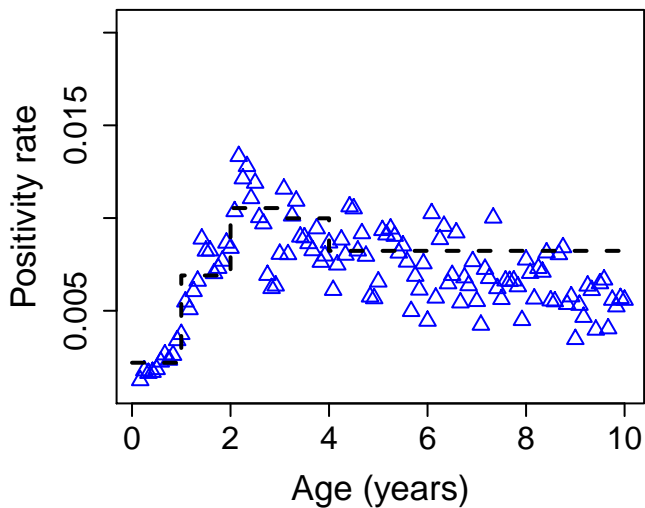

**2008**

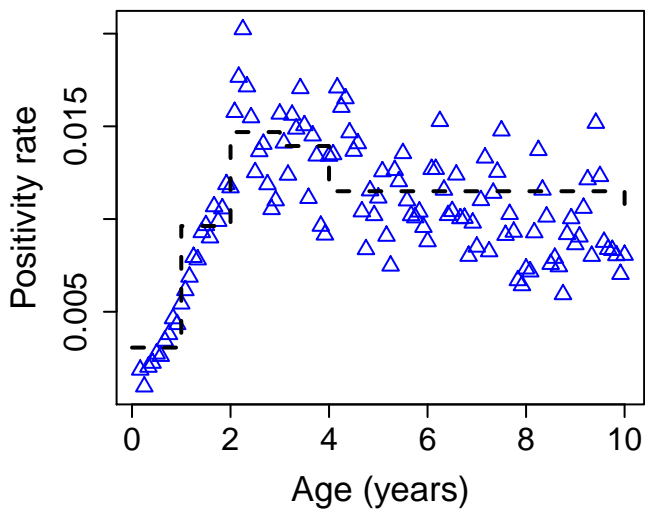

**2009**

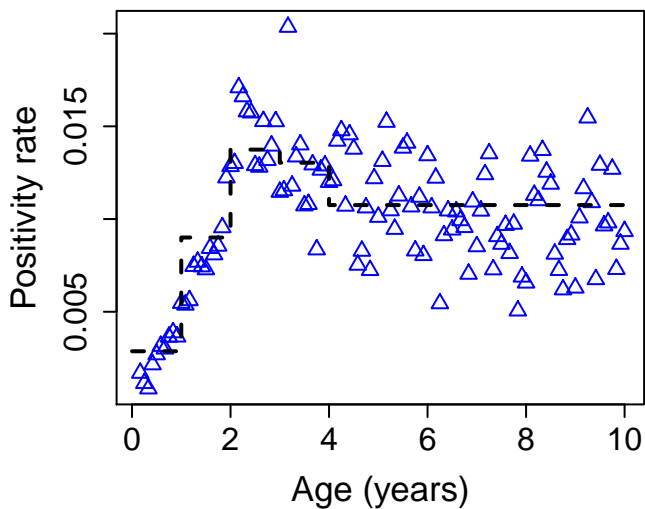

Supplement: Additional file 5 — Model fits for beef cattle for the years 2004 to 2009. Figures show data and model fit for the age/year-dependent model for beef cattle between 2004 and 2009. In each panel, the points are the data and the step function is the model fit from the age and year dependent model. [file 1297-9716-44-97-S5.pdf]

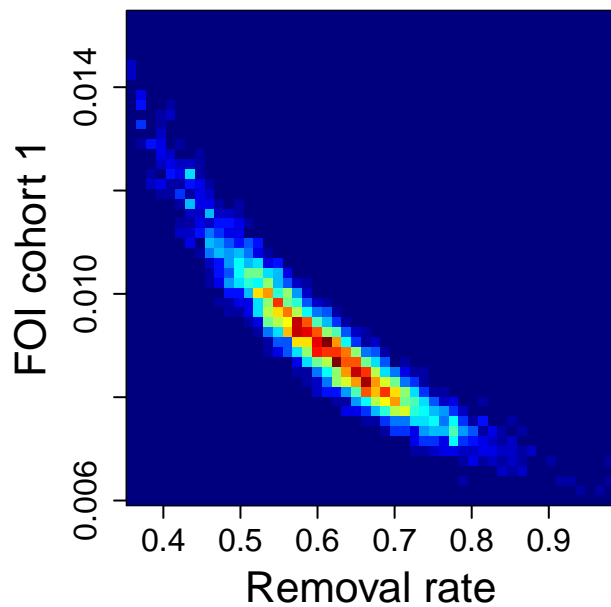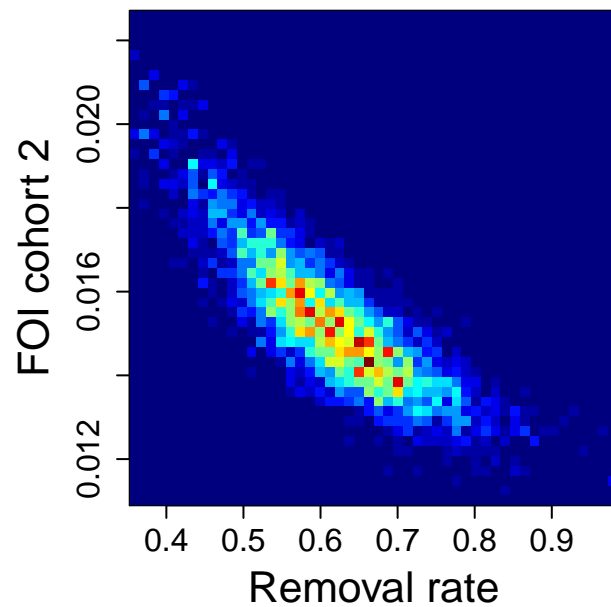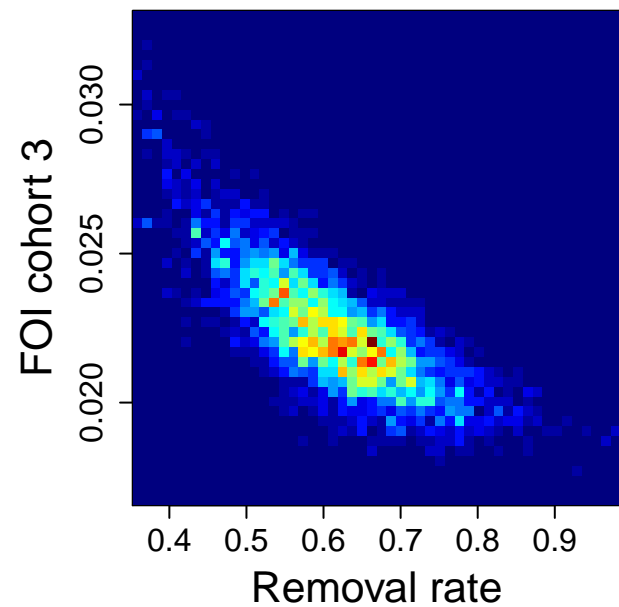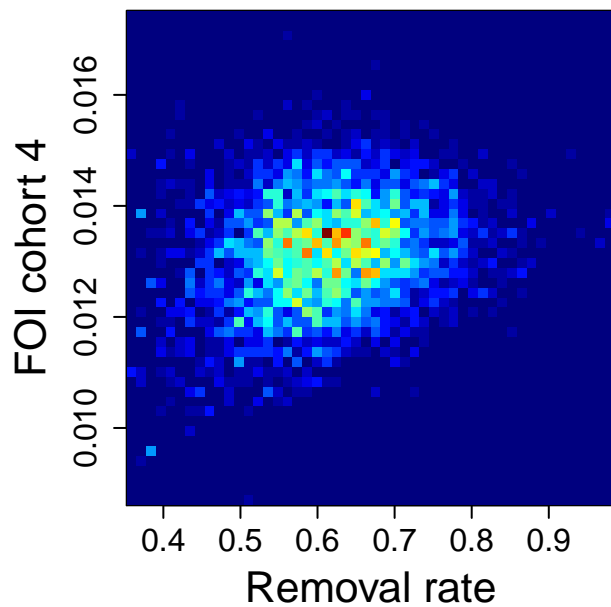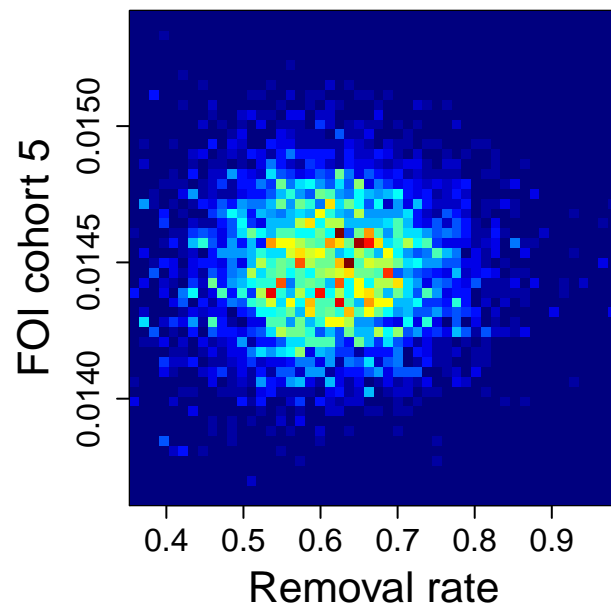

Supplement: Additional file 6 — 2D posterior densities illustrating the relationship between infection and removal rates. Figure illustrating the relationship between the removal rate and the age-specific infection rate. The areas of greatest probability are illustrated in red and lowest probability in blue. The force-of-infection (FOI) for each cohort is shown on the vertical axis and the removal rate on the horizontal axis. [file 1297-9716-44-97-S6.pdf]
